# Fine-Scale Population Admixture Landscape of Tai–Kadai-Speaking Maonan in Southwest China Inferred From Genome-Wide SNP Data

**DOI:** 10.3389/fgene.2022.815285

**Published:** 2022-02-17

**Authors:** Jing Chen, Guanglin He, Zheng Ren, Qiyan Wang, Yubo Liu, Hongling Zhang, Meiqing Yang, Han Zhang, Jingyan Ji, Jing Zhao, Jianxin Guo, Jinwen Chen, Kongyang Zhu, Xiaomin Yang, Rui Wang, Hao Ma, Le Tao, Yilan Liu, Qu Shen, Wenjiao Yang, Chuan-Chao Wang, Jiang Huang

**Affiliations:** ^1^ Department of Forensic Medicine, Guizhou Medical University, Guiyang, China; ^2^ State Key Laboratory of Cellular Stress Biology, School of Life Sciences, Xiamen University, Xiamen, China; ^3^ Department of Anthropology and Ethnology, School of Sociology and Anthropology, Institute of Anthropology, National Institute for Data Science in Health and Medicine, Xiamen University, Xiamen, China; ^4^ State Key Laboratory of Marine Environmental Science, Xiamen University, Xiamen, China; ^5^ Institute Of Rare Diseases, West China Hospital of Sichuan University, Chengdu, China

**Keywords:** fine-scale genetic structure, Tai–Kadai-speaking Maonan, admixture history, ethnolinguistic diversity, east Asian

## Abstract

Guizhou Province harbors extensive ethnolinguistic and cultural diversity with Sino-Tibetan-, Hmong–Mien-, and Tai–Kadai-speaking populations. However, previous genetic analyses mainly focused on the genetic admixture history of the former two linguistic groups. The admixture history of Tai–Kadai-speaking populations in Guizhou needed to be characterized further. Thus, we genotyped genome-wide SNP data from 41 Tai–Kadai-speaking Maonan people and made a comprehensive population genetic analysis to explore their genetic origin and admixture history based on the pattern of the sharing alleles and haplotypes. We found a genetic affinity among geographically different Tai–Kadai-speaking populations, especially for Guizhou Maonan people and reference Maonan from Guangxi. Furthermore, formal tests based on the *f*_*3*_/*f*_*4*_-statistics further identified an adjacent connection between Maonan and geographically adjacent Hmong–Mien and Sino-Tibetan people, which was consistent with their historically documented shared material culture (Zhang et al., iScience, 2020, 23, 101032). Fitted qpAdm-based two-way admixture models with ancestral sources from northern and southern East Asians demonstrated that Maonan people were an admixed population with primary ancestry related to Guangxi historical people and a minor proportion of ancestry from Northeast Asians, consistent with their linguistically supported southern China origin. Here, we presented the landscape of genetic structure and diversity of Maonan people and a simple demographic model for their evolutionary process. Further whole-genome-sequence–based projects can be presented with more detailed information about the population history and adaptative history of the Guizhou Maonan people.

## Introduction

Guizhou Province is localized in the Yunnan–Guizhou Plateau and has a mountainous environment. This region has rich archaeologically attested cultures, historically documented ethnic groups, and languages. Populations belonging to different language families, including Tai–Kadai, Hmong–Mien, and Sino-Tibetan families, permanently resided here. Archaeological findings associated with the Chengtoushan, Daxi, and Shijiahe sites supported that southern China was the original birthplace of rice agriculture and the homelands of many languages. Further direct evidence from both archeology and language supported that the prosperity of rice farming led to the formation of the ancestral populations of present-day Tai–Kadai-, Hmong–Mien-, Austroasiatic-, and Austronesian-speaking populations and their used languages (Diamond and Bellwood, 2003; Zhao, 2011; Stevens and Fuller, 2017). In addition, linguistic evidence not only demonstrated that there was a common origin between Tai–Kadai and Austronesian language but also revealed that the Tai–Kadai language shared more language components (language borrowing) with surrounding Hmong–Mien and Sino-Tibetan families (Edmondson, 1988; Edmondson, 1997; Lu, 2008), which was also confirmed *via* the genome-wide SNP data (He et al., 2020). Importantly, a recent published genetic analysis based on the whole-genome SNP data from Southeast Asians also revealed the complex divergence processes, which showed that Austroasiatic people diverged from mainland Chinese populations approximately 15 thousand years ago (kya), Austronesian people diverged from mainland Sinitic-speaking Han and Tai–Kadai-speaking Dai around 10 kya, and Cordilleran people split from indigenous Taiwanese people at eight kya (Larena et al., 2021). The divergence time between the ancestors of Hmong-Mien and Tai-Kadai people keep unknown, but they experienced massive interaction with each other and with the southward Northeast Asians (Huang et al., 2020; Yang et al., 2020; Wang et al., 2021c; Wang C.C. et al., 2021). The initial landscape of the population history of southern China has been characterized *via* evidence from genetics and linguistics, whose detailed genetic history and admixture process with its neighbors need to be further explored based on the high-density genome-wide SNP data, especially for some geographically and ethnolinguistically diverse and specific indigenous populations.

The linguistic survey has found that the Tai–Kadai language was widely distributed in Southeast Asia, including Zhuang-Dai, Dong-Shui, Li, and Ge-Yang language sub-branches (Edmondson, 1988; Liang and Zhang, 1996; Edmondson, 1997; Kutanan et al., 2019; Liu et al., 2020; Kutanan et al., 2021). Linguistic findings showed that Maonan is a subgroup of the Dong-Shui language, mainly distributed in Guizhou, Guangdong, and Guangxi provinces (Lu, 2008). Historians hold the opinion that Tai–Kadai people in South China are one of the indigenous population with a long history (Wang, 2004; Huang, 2016; Zhang, 2016), which is also evidenced *via* the linguistic documents and ancient DNA evidence (Edmondson, 1988; Liang and Zhang, 1996; Lipson et al., 2018; McColl et al., 2018; Liu et al., 2020; Kutanan et al., 2021). Direct documents from historic materials showed that the Maonan ethnic group is related to the modern Southern Chinese indigenous ethnic groups such as Bouyei, Mulam, and Gelao (Wang, 2004; Huang, 2016).However, the historical records for the origin of Maonan and the records of local Chronicles inscriptions and genealogies are unknown. From an archeological perspective (Ma et al., 2020), the presumed ancestral populations of modern Tai–Kadai and Hmong–Mien speakers are probably related to Daxi culture and Qujialing culture around the Yunnan–Guizhou Plateau. Therefore, the genetic investigation should be comprehensively carried out in areas with high ethnic and linguistic diversity to explore the genetic connection among modern ethnolinguistically different populations and the genetic interactions between Guizhou indigenous people with ancient northern and southern East Asians (Ning et al., 2020; Yang et al., 2020; Mao et al., 2021; Wang et al., 2021c; Wang C. C. et al., 2021).

Considering the importance of a comprehensive and deep genetic survey of South China, previous genetic studies based on forensic genetic markers have shed light on the basic genetic profile and demographic history among Tai–Kadai speakers from southern China in the past two decades. From the perspective of uniparentally inherited Y chromosome haplogroup and mitochondrial haplogroup, Chen et al. made a preliminary exploration focused on the forensic parameters and genetic structure of the Tai–Kadai-speaking populations in Guizhou and the population genetic relationship based on the short tandem repeat (STR) on the autosome and X/Y-chromosomes (Chen et al., 2018c). Further genetic studies had focused on the population admixture history and genetic diversity of Tai–Kadai-speaking Gelao and Bouyei by insertion/deletion polymorphisms (InDels) and ancestry-informative single-nucleotide polymorphism (AISNPs) (He et al., 2019a; He et al., 2019b; He et al., 2021b). The obtained research results showed a significant genetic interaction between Tai–Kadai- and Hmong–Mien-speaking populations (He et al., 2019a; He et al., 2019b; He et al., 2021b). However, these studies were conducted based on low-density genetic markers and were mainly focused on exploring forensic characteristics. The low resolution of low-density markers was restricted to provide a fine-scale population genetic structure that can show the detailed information for the population admixture, evolutionary, and adaptive history. Recent population genomic history characterization of ethnolinguistically diverse people in Guizhou, including Han, Chuanqing, Gejia, Dongjia, Xijia, Mongolian, Manchu, Bouyei, Sui, Tujia, Dong, and Gelao people, has provided new insights into their formation processes and complete landscape of genetic history (Lu et al., 2020; Chen et al., 2021; Bin et al., 2021; He et al., 2021a; Wang et al., 2021a; Wang et al., 2021b). However, the admixture history of another important Guizhou indigenous Maonan still remains unknown.

Maonan people used the Maonan language belonging to the Dongshui branch in the Tai–Kadai language family (Edmondson, 1988, 1997; Lu, 2008), which was widely distributed in Guizhou and Guangxi provinces. Historians supported that the Maonan people were one of the major descendants of ancient indigeneous tribes in coastal southern China, and that they were especially associated with the hanging coffin burial custom (Zhang et al., 2020). Based on the ancient DNA from the mitochondrial chromosome from historic hanging coffin people in Fujian, Guangxi, ancient people related to Tai–Kadai populations migrated westward from Fujian Wuyi Mount in the historical times and then across Southwest China to Southeast Asia (Zhang et al., 2020). Historical materials based on the hanging coffin customs also showed similar patterns of population migrations. Recent population genetic studies have included the Maonan people in Guangxi as the reference populations in the ancient DNA study. However, their fine-scale population history and their genetic relationship with the surrounding Hmong–Mien- and Sino-Tibetan-speaking populations have not been fully characterized, which are especially focused on the sharing of genome-wide haplotype data. Genetic studies of populations from the Yunnan–Guizhou Plateau regions have found enriched genetic diversity and complex mixed population genetic history (He et al., 2019a; He et al., 2019b; Zhang et al., 2019; Liu et al., 2021a; He et al., 2021b; Liu et al., 2021b; Chen et al., 2021). The detailed relationship between Maonan and modern and ancient neighboring populations needs to be characterized further. Thus, we conducted a genetic analysis based on the array-based genotyping of approximately 700 K SNPs in Tai–Kadai-speaking Maonan people in Guizhou to reconstruct its genetic diversity and evolutionary relationship with surrounding populations. Then, we merged our data with modern and ancient available East Asian data to explore their fine-scale population genetic structure and evolutionary history.

## Material and Methods

### Sample Collection and DNA Preparation

We collected saliva samples from 41 unrelated Maonan individuals in Pingtang County in Guizhou Province, Southwest China (Supplementary Figure S1). Participants whose parents and grandparents are indigenous people and reside in the sampling palaces at least three generations should have non-consanguineous marriage at the same ethnical group. The study was approved by the Medical Ethics Committee of Guizhou Medical University, and the recommendations provided by the revised Helsinki Declaration of 2000 were followed. All the participants signed written informed consent prior to participating in the study. We genotyped the genome-wide SNP data using the Infinium Global Screening Array, which included approximately 700 K SNPs and covered SNPs from the autosome, Y-chromosome, and mitochondrial DNA. We used a similar in-house commonly used standard to conduct quality control filter procedures (Wang et al., 2021b). Then, we merged the genome-wide data of 41 Guizhou Maonan individuals with previously published present-day and ancient East Asian and Southeast Asian populations from Human Origins (HO) and 1240 k datasets included in the Allen Ancient DNA Resource (AADR) and our recently published genome-wide SNP data based on the Illumina platform (Lu et al., 2020; Wang et al., 2020; Wang et al., 2021a; Liu et al., 2021b; Wang et al., 2021b; He et al., 2021c; Chen et al., 2021; Yao et al., 2021).

### Principal Component Analysis

We carried out principal component analysis (PCA) *via* the *smartpca* program of the EIGENSOFT v.6.1.4 package (Patterson et al., 2006) based on the merged Human Origin dataset. All default parameters were used with the additional parameter of lsqproject: YES, in which ancient DNA was projected based on the genetic landscape of the modern East Asians from Hmong–Mien, Tai–Kadai, Austronesian, Austroasiatic, and Sino-Tibetan speakers.

### ADMIXTURE Analysis

To prune the strong linkage disequilibrium, we first used PLINK tools (Chang et al., 2015) with the additional parameters (--indep-pairwise 200 25 0.4) to obtain the unlinked SNP data among Eurasian modern and ancient populations. Model-based clustering analysis was performed *via* ADMIXTURE (Alexander et al., 2009), and we ran ADMIXTURE with default parameters with the predefined ancestry sources or clusters ranging from K = 2 to 20. We assessed an optimal K value based on the lowest cross-validation error values using 10-fold cross-validation with different random seeds.

### Admixture-*f*_*3*_-Statistics and Outgroup-*f*_*3*_-Statistics

We used ADMIXTOOLS (Patterson et al., 2012) to compute *f*-statistic values and estimate standard errors by a block jackknife and default parameters. We used the *qp3Pop* program of EIGENSOFT to calculate the outgroup-*f*_*3*_-statistics in the form *f*_*3*_(Population 1, Population 2; Mbuti) using the default parameters, and this index was used for evaluating the shared genetic drift between Population 1 and Population 2 since their separation from the outgroup population of Mbuti. Then we also used the qp3pop to perform the admixture-*f*_*3*_-statistics in the form *f*_*3*_(Source 1, Source 2; Targeted population) to explore the admixture signals in Maonan samples with different East Asian and Southeast Asian ancestral source candidates. The value with |Z-score|>3 denoted that Source 1 and Source 2 could generate the potential admixture signal for the target population.

### *f*_4_ Statistics

We used the *qpDstat* program in ADMIXTOOLS (Patterson et al., 2012) with default parameters to assess whether W or X harbored more ancestry related to population Y in the *f*_*4*_ (W, X; Y, Outgroup), which can be used to determine the signals and directions of admixture, and the primary source of gene flow to Guizhou Maonan and other modern and ancient reference East Asians.

### *Pairwise qpWave and qpAdm* Estimation

We used *qpWave*/*qpAdm* as implemented in the ADMIXTOOLS (Patterson et al., 2012) package with default parameters and estimated standard errors to detect the minimum number of ancestral populations, and quantitatively estimate corresponding admixture proportions. We used ancient Northeast Asian-related ancestry as the northern sources and Guangxi- or Taiwan-related ancestry as the southern sources to perform the two population qpAdm model. 1500-year-old BaBanQinCen people are the major ancestral southern sources in our admixture models as it was reported as the direct ancestral sources of modern Tai–Kadai people (Wang et al., 2021c). BaBanQinCen was a meta-population, which comprised two individuals from the Balong site (BalongKD10 and BalongKD07), two individuals from the Banda site (BandaKD15 and BandaKD11), one individual from Qinchang (QinchangKD13 and QinchangKD14), and one individual from Cenxun (CenxunKP05). We used the Mbuti, Ust_Ishim, Kostenki14, Papuan, Australian, Mixe, MA1, Jehai, and Tianyuan as outgroups. We also conducted pairwise qpWave analysis among Tai–Kadai, Hmong–Mien, Sinitic, and ancient Guangxi people to explore their genetic homogeneity. Admixture times were estimated using ALDER with the sources from northern and southern East Asia (Loh et al., 2013).

### TreeMix

We ran TreeMix version 1.13 (Pickrell and Pritchard, 2012) to infer the patterns of population splits and admixtures between our target populations and multiple ancestral populations. First, we explored the genetic relationship between Maonan and 15 Chinese populations based on the Illumina array, which was also used in the following haplotype-based analysis. Second, we constructed the TreeMix-based phylogenetic tree among 39 populations to explore the genetic relationship with more reference populations.

### Haplotype-Based Fine-Scale Population Structure

We used SHAPEIT v2 (Browning and Browning, 2011) to phase the genome-wide data of Maonan and other Chinese populations in Guizhou and the neighboring regions. Then we conducted the ChromoPainter and FineSTRUCTURE analysis (Hellenthal et al., 2014) to explore the coancestry matrix. We also used R packages implemented in the FineSTRUCTURE to perform the PCA analysis and explore the phylogenetic relationship of studied individuals and populations.

### Uniparental Haplogroups

Based on this Illumina array, we genotyped the lineage-informative SNPs (LISNPs) in mitochondrial DNA and Y-chromosome. The haplogroup assignment was used as the in-house manuscripts followed by our recent publications (Lu et al., 2020; Wang et al., 2020; Wang et al., 2021a; Liu et al., 2021b; Wang et al., 2021b; He et al., 2021c; Chen et al., 2021; Yao et al., 2021).

## Results

### General Structure Inferred From ADMIXTURE and PCA

**FIGURE 1:**
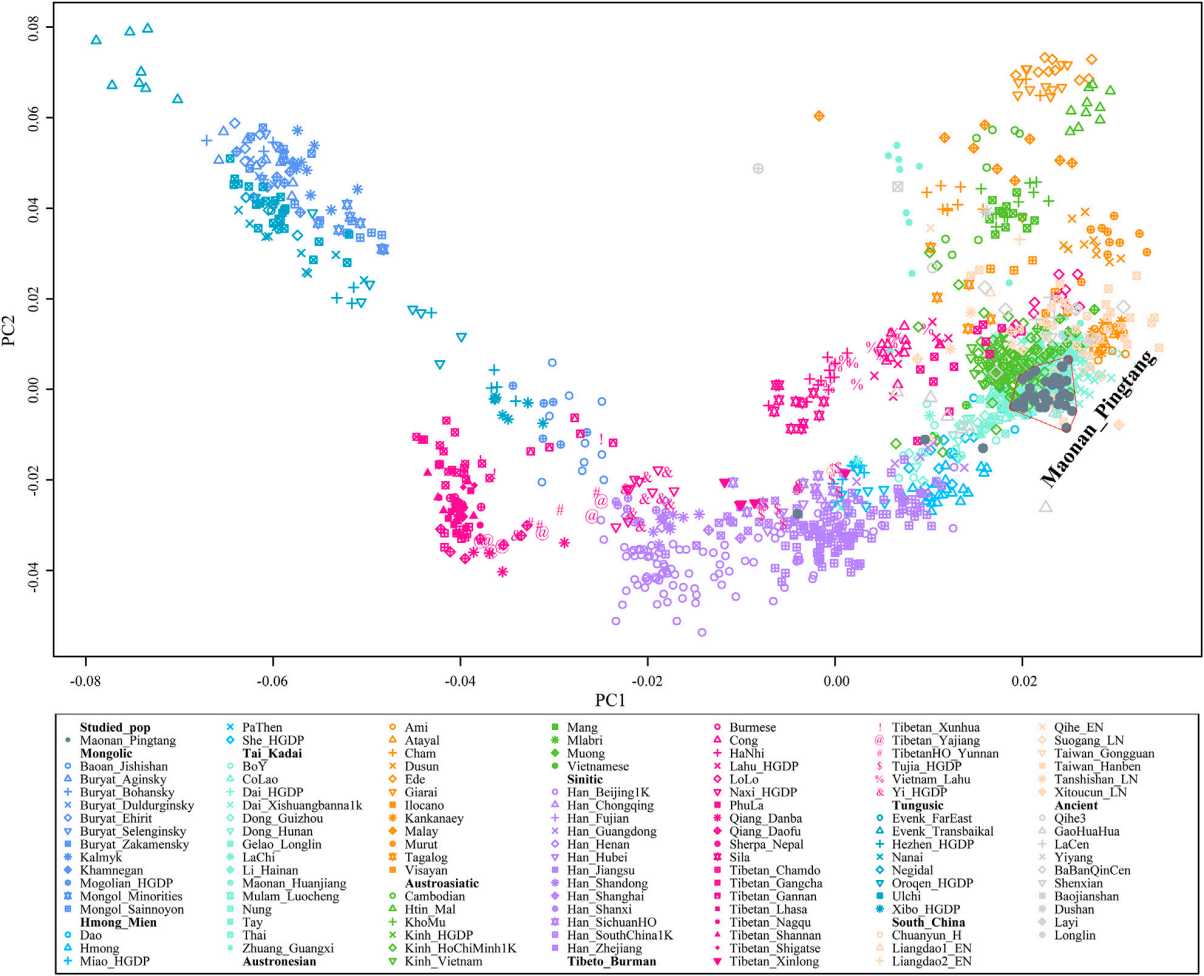
Principal component analysis between Maonan and other modern and ancient references of East Asians.

We generated genome-wide data in approximately 700,000 SNPs for 41 Maonan individuals from Guizhou Province, Southwest China. We first merged our data with modern and ancient published populations from the Human Origins dataset. Then we carried out a principal component analysis (PCA) to understand the general patterns of relatedness between Guizhou Maonan and reference populations (Figure 1). We observed three major genetic clines: the northern cline consisting of Mongolic- and Tungusic-speaking populations, the southern cline comprising Hmong–Mien-, Tai–Kadai-, Austroasiatic-, and Austronesian-speaking populations, and the Sino-Tibetans comprising Sinitic- and Tibeto–Burman-speaking populations, which was located at an intermediate position between the northern cline and the southern cline. We projected publicly available data of ancient individuals from China into modern PC plots. Our studied Tai–Kadai-speaking Maonan population all overlapped with modern Tai–Kadai populations. To gain further insight into the genetic architecture of Guizhou Maonan, we further focused on the genetic backgrounds of Tai–Kadai and other southern modern and ancient East Asians (Supplementary Figure S2). We observed that our studied group partially overlapped with previously published Austroasiatic- and Hmong–Mien-speaking populations.

**FIGURE 2:**
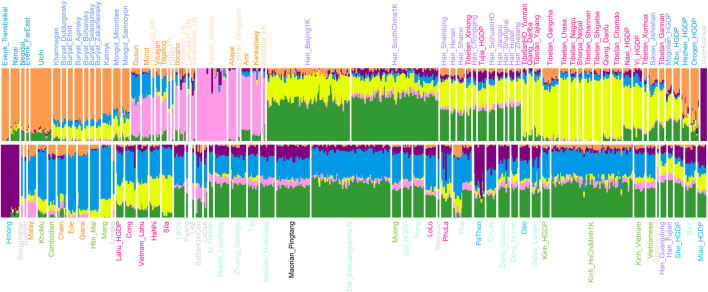
Model-based ADMIXTURE analysis showed the admixture composition of Eurasians.

We carried out the model-based ADMIXTURE clustering analysis to dissect ancestral components and genetic similarity of our studied group with geographically close ancient and present-day populations. We used cross-validation to identify an “optimal” number of clusters (K = 6) (Figure 2). At optimal K = 6, we observed four specific ancestral components in our studied Pingtang Maonan in Guizhou Province: the ancestry maximizing in this cluster is ubiquitous in modern Sinitic-speaking populations (dark green), with the second component maximized in Austroasiatic-speaking populations (dark blue). The remaining ancestry component was maximized in Hmong–Mien- (dark purple) and Austronesian-speaking populations (dark pink). Hmong–Mien-related ancestry component was maximized in historical GaoHuaHua individuals and found at the highest proportions in Hmong. Austronesian-related ancestry component was maximized in ancient and modern Austronesian Taiwanese with a high proportion in earlier Fujian Neolithic individuals (Taiwan_Hanben/Taiwan_Gongguan) and found at the highest proportions in Atayal. We found that our studied Pingtang Maonan is genetically like the other Tai–Kadai-speaking populations, in which both harbored similar patterns of ancestry components.

### Phylogenetic Relationship Sharing Alleles and Sharing Haplotypes

**FIGURE 3:**
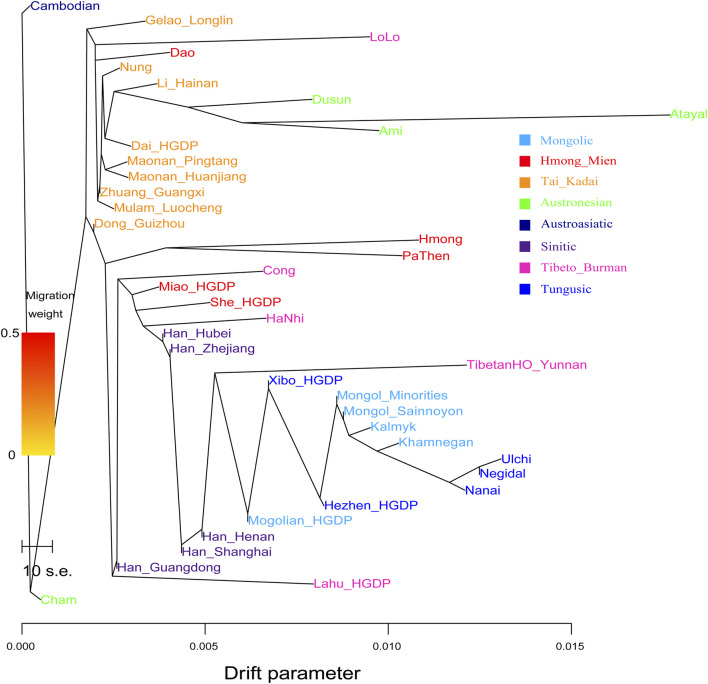
Phylogenetic relationship among East Asians showed the close genetic relationship between Maonan and Chinese Tai–Kadai and Hmong–Mien people.

The estimated pairwise Fst genetic distances showed the close genetic relationship between Maonan and geographically close Tai–Kadai-speaking populations (Sui and Maonan people in Guangxi), which is consistent with the identified genetic affinity based on the outgroup-*f*_*3*_ values. Based on the Illumina Array dataset, we further explored the phylogenetic relationship with admixture among 16 Chinese populations belonging to Sinitic, Tungusic, Mongolic, and Tai–Kadai people (Supplementary Figure S3). We found that Pingtang Maonan clustered with the Sandu Sui people and following closed with Manchu and Mongolian people in Guizhou Province. We did not identify gene flow events into Maonan or from Maonan influx to other reference populations. In addition, to explore the genetic relationship with more reference populations in the maximum-likelihood–based TreeMix tree, we also reconstructed a tree among 39 East Asians (Figure 3). We found that the Northeast Asians clustered closely with each other, including Tungusic-, Mongolic-, and Sino-Tibetan-speaking populations. Southeast Asians also clustered with each other, including Tai–Kadai and Austronesian people. The genetic affinity between Maonan people and other Tai–Kadai-speaking populations was once again confirmed here, including Dai, Li, Zhuang, and Mulam, and in other Austronesian-speaking populations (Ami and Atayal). This identified genetic phylogenetic relationship further confirmed the close genetic relationship between Tai–Kadai and Austronesian people, which was consistent with recent linguistic similarities.

**FIGURE 4:**
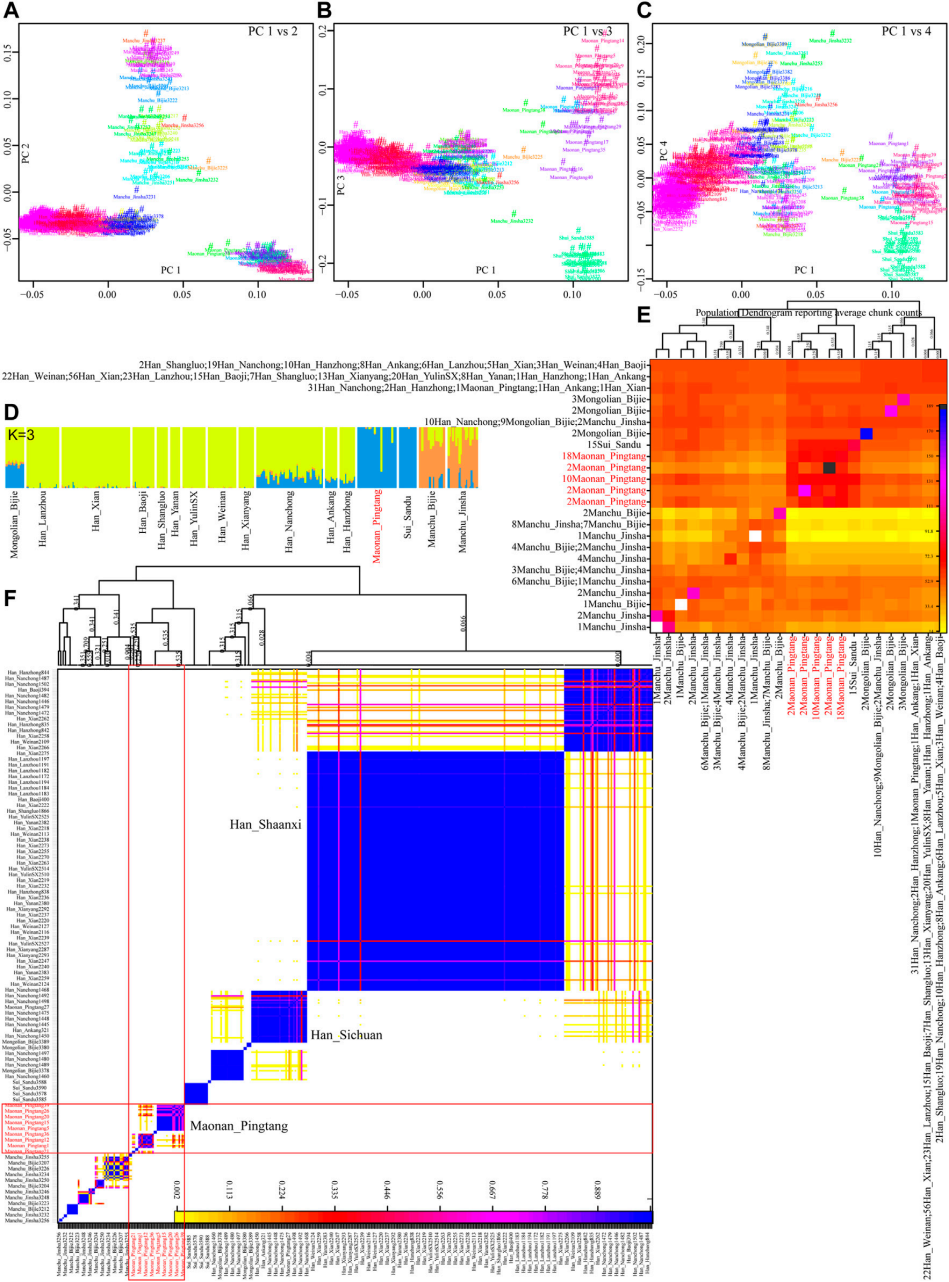
Fine-scale genetic structure among Maonan and other Chinese populations. **(A–C)** Principal component analysis based on the coancestry matrix. **(D)** Model-based ADMIXTURE results showed the three-ancestry component among the used Chinese populations. **(E,F)** Clustering patterns of Chinese populations or individuals based on the pairwise coincidence matrix inferred from the coancestry matrix.

**FIGURE 5:**
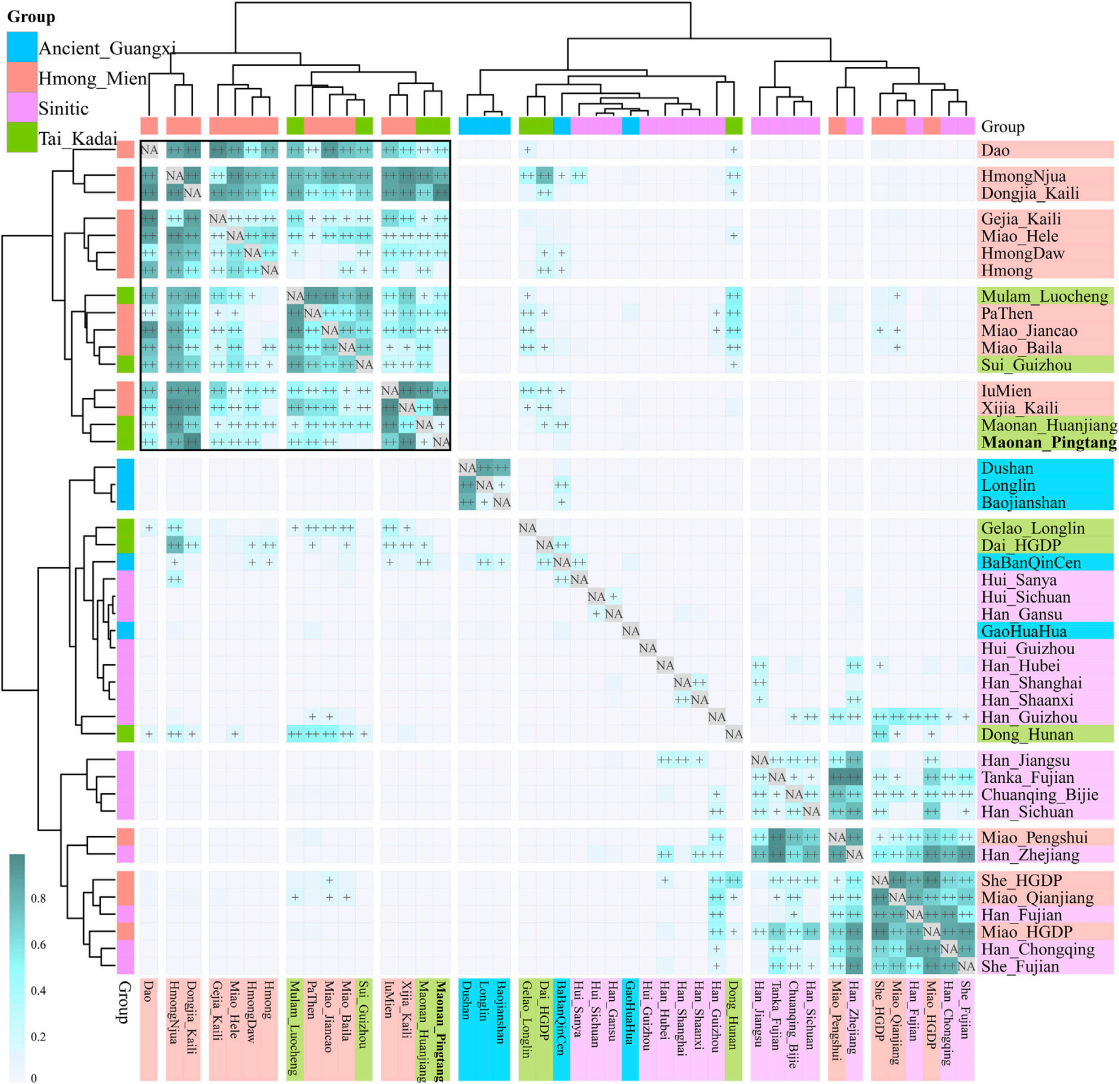
Pairwise qpWave results showed the genetic homogeneity between Maonan and Tai–Kadai and Hmong–Mien people.

We further explored the fine-scale population structure between Maonan people and other East Asians based on the patterns of the sharing haplotypes. PCA based on the coancestry matrix showed that Han Chinese clustered together and separated from Pingtang Maonan people and Guizhou Manchu and Mongolic people, which showed that the Maonan people had a different genetic history compared with Han Chinese populations (Figure 4A–C). Model-based ADMIXTURE results with three predefined ancestral sources further confirmed the genetic differentiation among Han Chinese, Manchu, and Maonan, who shared their unique ancestry component in Figure 4D. We also explored the genetic relationship among these populations based on the individual-level and population-level pairwise coincidence matrix estimated from the coancestry data (Figure 4E–F). We found that Maonan people clustered as a separated clade and then clustered with Manchu people. We finally explored the genetic heterozygosity and homogeneity among Tai–Kadai, Hmong–Mien, and Sinitic people using pairwise qpWave analysis. We found the statistically non-significant values (p_rank0>0.05) between Maonan and other Tai–Kadai populations, as well as the geographically close Hmong–Mien people. These observed patterns suggested Maonan had a relatively close relationship with other Guizhou populations when a distant outgroup was used here (Supplemntary Table S1). Indeed, we found that Maonan, Hmong–Mien, and Tai–Kadai people shared similar patterns of the distribution of the p_rank0 values, and they clustered together and formed one clade in Figure 5.

### Shared Genetic Drift Inferred From *F*-Statistics

To explore the relationship between the investigated population and reference populations, we measured allele sharing and admixture signals *via* outgroup-*f*_*3*_ and admixture-*f*_*3*_-statistics. We performed outgroup-*f*_*3*_ statistics in the form of *f*_*3*_(*X, Maonan_Pingtang; Mbuti)*, and found that Guizhou Maonan shared more genetic drift with southern Chinese populations, especially for Hmong–Mien- (e.g., Yao, Gejia, and Dongjia) and Tai–Kadai-speaking populations (e.g., Mulam, Zhuang, and Dong) (Supplementary Table S2A). Then we used admixture-*f*_*3*_ statistics of the form *f*_*3*_(*Source 1, Source two; Maonan_Pingtang)* to model possible sources for Maonan_Pingtang people (Supplementary Table S2B). However, we did not observe admixture signals (Z-scores less than -3) significantly in the Maonan_Pingtang when we used different East Asian and Southeast Asian ancestral source candidates.

To further explore the differentiation between the Maonan_Pingtang and other East Asian populations, we performed the *f*_*4*_(*Reference population 1, Studied population; Reference population 2, Mbuti*)*.* The identified statistically non-significant *f*_*4*_-values (absolute Z-scores less than 3) in *f*_*4*_(*Maonan_Huanjiang, Maonan_Pingtang; East Asians, Mbuti*) indicated that Maonan_Pingtang and Maonan_Huanjing have a close genetic relationship (Supplementary Table S3A) compared with other reference populations. Focused on other Tai–Kadai-speaking populations, we observed significantly negative *f*_*4*_-values in *f*_*4*_(*Dai/Zhuang_Guangxi, Maonan_Pingtang; East Asians, Mbuti*), which suggested that Guizhou indigenous populations and Hmong–Mien-speaking populations shared more alleles with Maonan_Pingtang than other Tai–Kadai-speaking populations (Supplementary Table S3B). Among Austronesian-speaking populations, the observed significant negative *f*_*4*_ statistics in the form *f*_*4*_(*Atayal/Ami, Maonan_Pingtang; East Asians of Hmong–Mien- and Tai–Kadai-speaking population, Mbuti)* in Supplementary Table S3C showed that Pingtang Maonan shared more alleles with Hmong–Mien- and Tai–Kadai-speaking populations compared with modern Austronesian people. Further comparative ancient DNA evidence demonstrated that Maonan_Pingtang also harbored more ancestry related to Hmong–Mien- and Sinitic-speaking populations when we used Neolithic to Iron Age from Fujian and Taiwan as reference population 1 in the form *f*_*4*_(*ancient southeastern East Asians, Maonan_Pingtang; East Asians, Mb*uti) (Supplementary Table S3D).

To directly compare the genetic relationship between Maonan- and Hmong–Mien-speaking populations, we used Hmong–Mien-speaking populations in Vietnam and Guizhou as reference population one in the form *f*_*4*_(*Hmong–Mien-speaking populations, Maonan_Pingtang; East Asians, Mbuti).* We observed significantly negative *f*_*4*_-values here, indicating that Maonan_Pingtang shared more alleles with Tai–Kadai- and Austronesian-speaking populations (Supplementary Table S3E,F) than with Hmong–Mien-speaking populations. To explore the genetic relationship between the studied population and the ancient populations in Guangxi, we used GaoHuahua, Longlin, Baojianshan, and BaBanQinCen as reference population 1. We observed that Guizhou Maonan shared more alleles with Hmong–Mien- and Sino-Tibetan–speaking populations than GaoHuahua, Longlin, and Baojianshan, as we observed significantly negative *f*_*4*_-values in the form of *f*_*4*_(*GaoHuahua/Longlin/Baojianshan, Maonan_Pingtang; East Asians, Mbuti*)*,* which suggests studied population was influenced by gene flow from the north (Supplementary Tables S3G–I). We have not observed significant negative *f*_*4*_-values in *f*_*4*_(*BaBanQinCen, Maonan_Pingtang; East Asians, Mbuti*), indicating that Maonan_Pingtang shared more alleles with northern populations than BaBanQinCen in Guangxi. We observed BaBanQinCen shared more alleles with other ancient populations than Maonan_Pingtang *via* significant positive *f*_*4*_-values in the form of *f*_*4*_(*BaBanQinCen, Maonan_Pingtang; ancient East Asians, Mbuti)* (Supplementary Table S3J). We observed significant negative *f*_*4*_-values in *f*_*4*_(*Austroasiatic-speaking populations, Maonan_Pingtang; East Asians, Mbuti*), which showed that Sinitic-, Tai–Kadai-, and Hmong–Mien-speaking populations from southern China shared more alleles with Maonan_Pingtang than Austroasiatic-speaking populations (Supplementary Table S3K). Significant negative values were observed in the *f*_*4*_(*southern Tibeto-Burman-speaking population, Maonan_Pingtang; East Asians, Mbuti*), it was shown that Tai–Kadai-, Austronesian-, and Hmong–Mien-speaking populations shared more alleles with studied population than southern Tibeto–Burman-speaking populations (Supplementary Table S3L). We found that Maonan_Pingtang harbored more Southeast Asian–related ancestry than ancient Yellow River farmers *via* significant negative *f*_*4*_-values in *f*_*4*_(*ancient Yellow River farmers, Maonan_Pingtang; Southeast Asians, Mbut*i) (Supplementary Table S3M).

### Admixture Modeling and Estimates of Admixture Times

**FIGURE 6:**
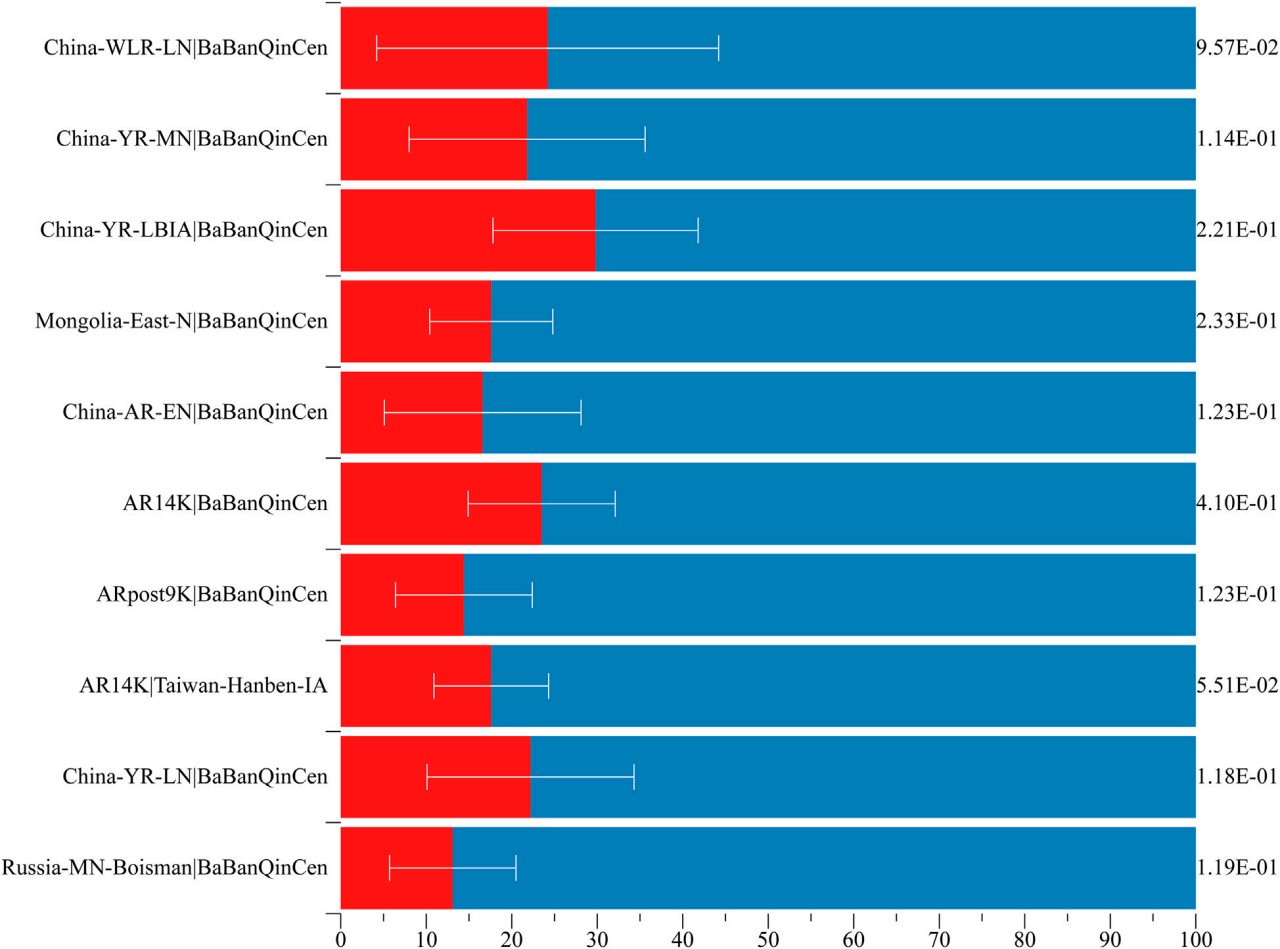
Two-way admixture models showed that both Northeast Asians and Southeast Asians contributed to the formation of Maonan people.

To explore the genomic formation of Guizhou Maonan people, we applied *qpWave*/*qpAdm* methods to model the minimum number of ancestry sources and evaluated the corresponding ancestry coefficients. We used ancient northern populations (Russia_MN_Boisman, China_YR_LN, AR14K, Chokhopani, ARpost9K, AR14K, China_AR_EN, Mongolia_East_N, China_YR_LBIA, China_YR_MN, and China_WLR_LN) as the northern ancestral sources and ancient southern populations (Taiwan_Hanben_IA, Guangxi_BaBanQinCen) as the southern ancestral sources to estimate the admixture proportions. Here, BaBanQinCen was the representative ancestral source in Guangxi Province, where many Tai–Kadai people lived. When we used ancient Yellow River farmers as the northern source, Southeast Asian ancestry related to the BaBanQinCen in Maonan people spanned from 70.2 to 86.9% (Figure 6, Supplementary Table S4). Date estimates with southern and northern East Asians further revealed that these identified north-to-south admixture events occurred in different historical times (Supplementary Table S5), which is consistent with genetically attested and historically documented southward population movements (Yang et al., 2020; Wang et al., 2021c; Wang C. C. et al., 2021).

### Uniparental Admixture History of Maonan People

Southern China was the originated center of many dominant uniparental lineages of southern Chinese indigenous populations and Southeast Asians (Ke et al., 2001; Wen et al., 2004; He et al., 2020; Sun et al., 2021). Sun et al. provided a higher-resolution phylogeny of O1a-M119 and found this founding lineage widely existed in modern Austronesian-, Tai–Kadai-speaking populations, and southern Han Chinese, suggesting that O1a-M119 lineage was the common lineage of these populations, and the diversified sub-lineages of O1a-M119 were their unique downstream paternal lineages (Sun et al., 2021). Other population genetic studies of Southeast Asians also identified other paternal founding lineages, including O1b1a1a-M95 and O2-M122 (Wen et al., 2004). Similarly, the complex landscape of maternal lineages in South China was also identified in the previous control-region or whole mitochondrial sequencing projects, including D4, B4, and M7 (Li et al., 2019; Mengge et al., 2020). Here, we also identified that northern and southern paternal and maternal lineages contributed to the uniparental gene pool of Guizhou Maonan people (Supplementary Table S6). We observed eight paternal Y-chromosome lineages (O1a1a2a1, O1b1a1a1a1a1a1b, O2a2b1a1a5a2, D1a1a2a2∼, O1b1a1a1b, O1b1a1a1a1a1a1a1a1, O2a1c2, and O1b1a1a1a1a1a1a2-Z24131) with the frequencies ranging from 0.0435 to 0.6957 among 23 males, in which haplogroup of O1b1a1a1a1a1a1a2-Z24131 was observed in 16 individuals. Our results showed that Southeast Asian-dominant paternal lineage of O1b1a1a1a1a1a1a2 was the founding lineage of Tai–Kadai-speaking Maonan people. O1b1a1a* (O-M95*) was previously evidenced to contribute much to the paternal gene pool of populations from South China, Thailand, and Laos (Kutanan et al., 2019). O1b1a1a1a* was also evidenced has experienced significant population expansions in the Neolithic period in Tai–Kadai and Austroasiatic populations. Thus, combined with the identified unique population structure of Maonan people based on the autosomal SNPs and this identified specific lineage of O1b1a1a1a1a1a1a2 with high frequency, Maonan people could be treated as the most representative inland Tai–Kadai-speaking population of southern ancient indigenous populations with the relatively minor genetic influence of southward Han Chinese expansion. Our results also suggested that the genetically documented marriage pattern was consistent with that of the culturally documented customers of patrilocality, which is also evidenced *via* the marriage pattern among Tai–Kadai people in Southeast Asians (Kutanan et al., 2019). In addition, we assigned all 41 maternally inherited mtDNA lineages into 25 terminal lineages with frequencies ranging from 0.0244 to 0.0732 (B4a1e, B5a1c1, F1a, F1c, F3b, and B4), which were observed frequently in both northern and southern East Asians.

## Discussion

Guizhou Province is rich in ethnolinguistic and cultural diversity (Wang et al., 2021a). Previous genetic studies have investigated the general landscape of genetic variations of Guizhou populations based on the autosomal, X/Y-chromosomal short tandem repeats (STRs), and ancestry-informative SNPs (Chen et al., 2018a; Chen et al., 2018b; Chen et al., 2018c; Chen et al., 2018d; Chen et al., 2019a; Chen et al., 2019b; He et al., 2021b). Archaeologically attested Daxi, Qujialing, and Shijiahe people were occupied in what is now Guizhou Province, and the present Guizhou region was occupied by Sino-Tibetan–speaking (Han, Yi, and others), Tai–Kadai-speaking (Gelao, Bouyei, Dong et al.), and Hmong–Mien-speaking populations (Miao, She, and Yao). Recent ancient DNA studies from the Yellow River Basin in Northeast Asia, Fujian, and Guangxi provinces from Southeast Asia also found that the bi-directional north-to-south population movements have shaped the genetic landscape of East Asians (Ning et al., 2020; Yang et al., 2020; Wang et al., 2021c; Mao et al., 2021; Wang C. C. et al., 2021). Demographic modeling of central Chinese populations, including Han and Tujia, also showed the genetic influences from both the northern millet farmers and southern rice farmers (He et al., 2021a). The Tai–Kadai language family and their corresponding people widely existed in Guizhou and surrounding regions; however, the fine-scale genetic structure of this linguistically specific population still needs to be further explored.

We genotyped the genome-wide SNP data from 41 Tai–Kadai-speaking Maonan people and explored their genetic origin, admixture history, and phylogenetic relationship with surrounding populations. Descriptive analysis based on the PCA and ADMIXTURE analyses showed that the Guizhou Maonan people and Guangxi Maonan people had the closest genetic relationship and shared the most genetic affinity, suggesting their common origin and admixture history. The genetic affinity within the population among Tai–Kadai-speaking populations was also evidenced by the Dong and Bouyei based on the Affymetrix-based array data (Wang et al., 2021a). In addition, population genetic analysis based on the forensic genetic markers (STR and Indels) also revealed the genetic affinity between geographically different Tai–Kadai-speaking populations rather than populations from other language families (Chen et al., 2018c; He et al., 2019a; He et al., 2019b). Interestingly, we also identified the genetic affinity between Maonan and Hmong–Mien-speaking populations in Guizhou Province among the non-Tai–Kadai-speaking populations based on the observed non-statistically significant *f*_*4*_-statistics in the form *f*_*4*_(*Maonan, Hmong–Mien-speaking Gejia/Dongjia/Xijia; other reference Asians populations, Mbuti*) and statistically negative *f*_*4*_-values in *f*_*4*_ (*Asian reference populations, Maonan; Hmong–Mien, Mbuti).* Our results were consistent with previous reports based on the forensic markers. He et al. explored the genetic diversity and forensic features of Guizhou Tai–Kadai-speaking Gelao people and identified the population interplay between Gelao and neighboring Hmong–Mien-speaking populations (He et al., 2019b). Our identified extensive genetic admixture between Hmong–Mien and Tai–Kadai people suggested that there was no clear genetic barrier between geographically close but linguistically different ethnic groups, which suggested that they have experienced extensive population interaction although initially they were of independent origin.

Linguistic interaction between Tai–Kadai and Sino-Tibetan languages was widely documented (Edmondson, 1988; Edmondson, 1997; Lu, 2008). Population interaction between Maonan- and Sino-Tibetan-speaking populations was also identified in our genetic study, which is consistent with other recently published population genetic investigations. He et al. recently explored the fine-scale genetic structure of four Guizhou Han populations and found their extensive admixture with Guizhou indigenes (Wang et al., 2021b). In addition, Wang et al. studied the genetic admixture of Guizhou culturally unique Hui people and found their connection between indigenous Han people (Wang et al., 2020). Other genome-wide SNP-based genetic analyses focused on Guizhou officially unrecognized Chuanqing people also found their genetic affinity with geographically close Han populations (Lu et al., 2020). Additionally, we found a close genetic relationship between Maonan and Guizhou Hans in the *f*_*3*_/*f*_*4*_-statistics and the TreeMix-based maximum-likelihood–based phylogenetic tree, which suggested their recent admixture process. The shared ancestry between Guizhou Maonan and Han people in ADMIXTURE and their qualitative indices was consistent with the shared cultural background between present-day Guizhou Han and Maonan people.

More and more ancient genomes in the surrounding regions of Guizhou Province were reported recently, especially important ancestry sources of the possible ancestor of Tai–Kadai-speaking populations (BaBanQinCen) and a possible ancestor of Hmong–Mien-speaking populations (GaoHuaHua). BaBanQinCen was one meta-population from four archeological sites that lived in Guangxi Province 2000 years ago, and GaoHuaHua was also a meta-population from three archaeological sites that lived in Guangxi around 1,500 years ago (Wang et al., 2021c). Here, we found that Maonan people shared the most genetic affinity with ancient Guangxi historic BaBanQinCen, which was recently genetically attested as the direct ancestor of Guizhou Tai–Kadai people (Wang et al., 2021c). Outgroup-*f*_*3*_-statistics and shared ancestry inferred from *f*_*4*_-statistics further confirmed the closest genetic connection between Maonan and BaBanQinCen compared with other Guizhou historic people (GaoHuaHua, Layi et al.) and prehistoric Longlin, Dushan, and Baojianshan people. Among all reported ancient Northeast Asians, including the inland and coastal Neolithic Northeast Asians from Shandong, Henan, Shaanxi, Gansu, Inner Mongolia in the Yellow River Basin, and Neolithic Siberian, we found a close genetic connection between Maonan and Bronze Age to Iron Age people from Henan Province, which suggested Maonan people might have obtained gene influxes from them. Indeed, we obtained statistically negative *f*_*4*_-values in the *f*_*4*_(*Guangxi historic people, Maonan; Yellow River millet farmers, Mbuti),* which directly evidenced that compared with the genetically attested ancestors of Tai–Kadai-speaking populations from the Guangxi region, Maonan people shared additional genetic materials from Northeast Asians. This identified admixture process was further confirmed *via* the qpAdm-based two-way admixture models with one source from Guangxi historic people and the other sources from Northeast Asians. We also found that two-way admixture models of Hanben-Northeast Asians can also well-fit the genetic composition of studied Maonan, which suggests the genetic influence from the southeastern coastal Fujian ancient people in the gene pool of inland Tai–Kadai-speaking Maonan people. As we know, Tai–Kadai-speaking populations were widely distributed in South China, including Guizhou, Guangxi, and Hainan. Previous genetic analysis from Hainan Province also reported a relatively isolated genetic structure of the Hainan Tai–Kadai-speaking Hlai people (He et al., 2020). Thus, dense sampling of Tai–Kadai-speaking populations and obtaining their whole-genome sequencing data would help to characterize the complete genetic admixture landscape of Chinese Tai–Kadai-speaking populations.

## Conclusion

We reported the first batch of genome-wide SNP data of Tai–Kadai-speaking Maonan people from Guizhou Province and comprehensively explored genetic structure, origin, and admixture processes based on the descriptive analyses (PCA and ADMIXTURE) and qualitative measures (*f*-statistics, *qpAdm*). Results from PCA and ADMIXTURE showed a close genetic relationship between Maonan and other geographically different Tai–Kadai-speaking populations, especially for the closest relationship between Guizhou Maonan and Guangxi Maonan. No-admixture signatures were identified *via* admixture-*f*_*3*_ statistics showed the unique genetic structure of Maonan people compared with geographically close Han people. Further analysis based on the outgroup-*f*_*3*_ statistics and *f*_*4*_-based analysis showed a close relationship between Maonan and Guizhou Sino-Tibetan and Hmong-speaking populations, as well as a close connection between Guangxi historic people and Guizhou Tai–Kadai-speaking populations, suggesting their admixture history with the sources from surrounding regions. The well-fitted two-way admixture models with ancient northern and southern East Asians demonstrated that Tai–Kadai-speaking populations derived primary ancestry related to 1500-year-old Guangxi BaBanQinCen people and additional genes from Northeast Asia.

## Data Availability

The datasets presented in this study can be found in online repositories. The names of the repository/repositories and accession number(s) can be found below: https://zenodo.org/record/5701604, 10.5281/zenodo.5701604.
