# Supplementary figures and images for: Fine-Scale Population Admixture Landscape of Tai–Kadai-Speaking Maonan in Southwest China Inferred From Genome-Wide SNP Data
Source: Front Genet. 2022 Feb 17;13:815285. doi: 10.3389/fgene.2022.815285 (PMC8891617; doi:10.3389/fgene.2022.815285)

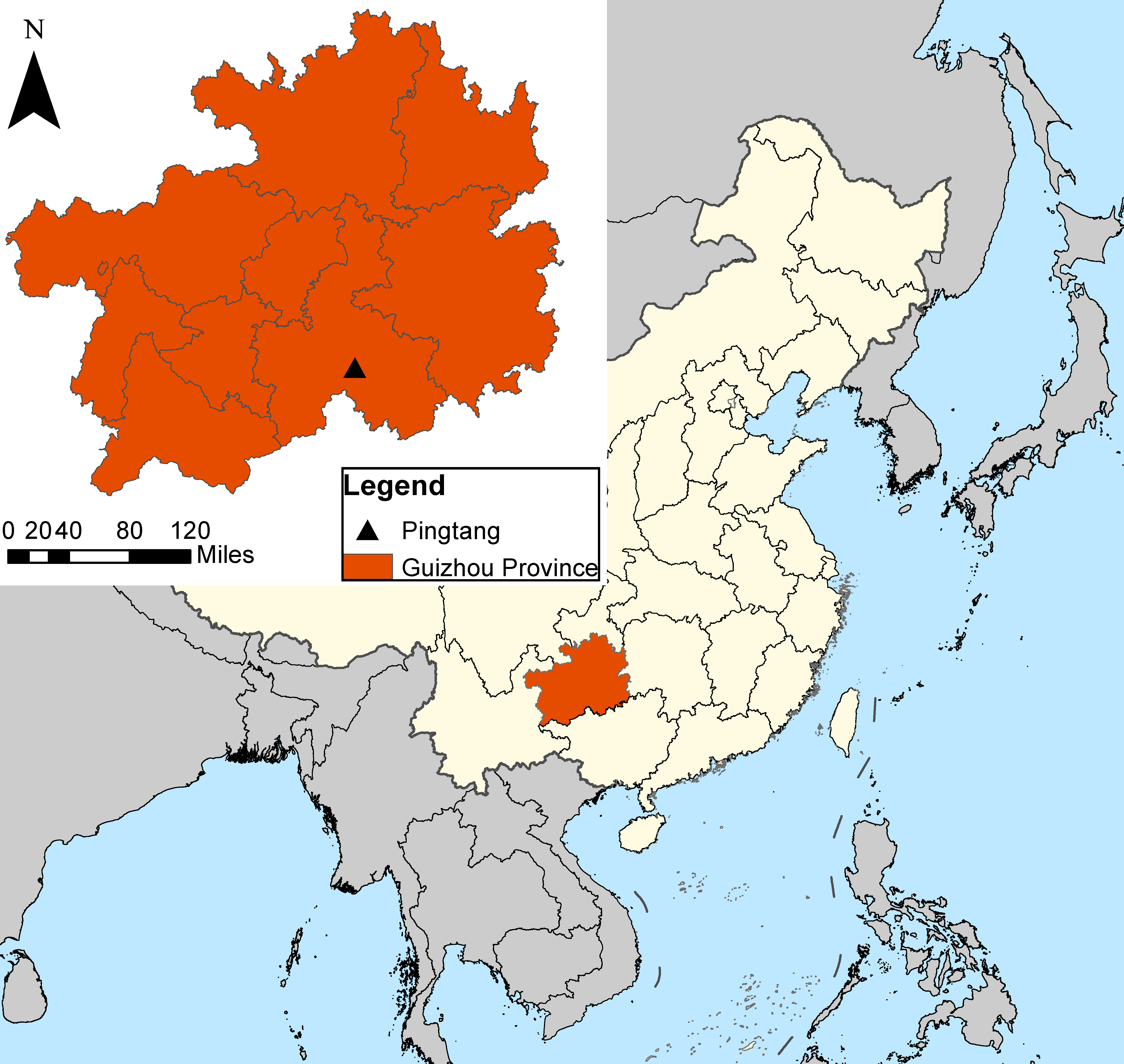

Supplement: Supplementary file 2 [file Image1.TIFF]

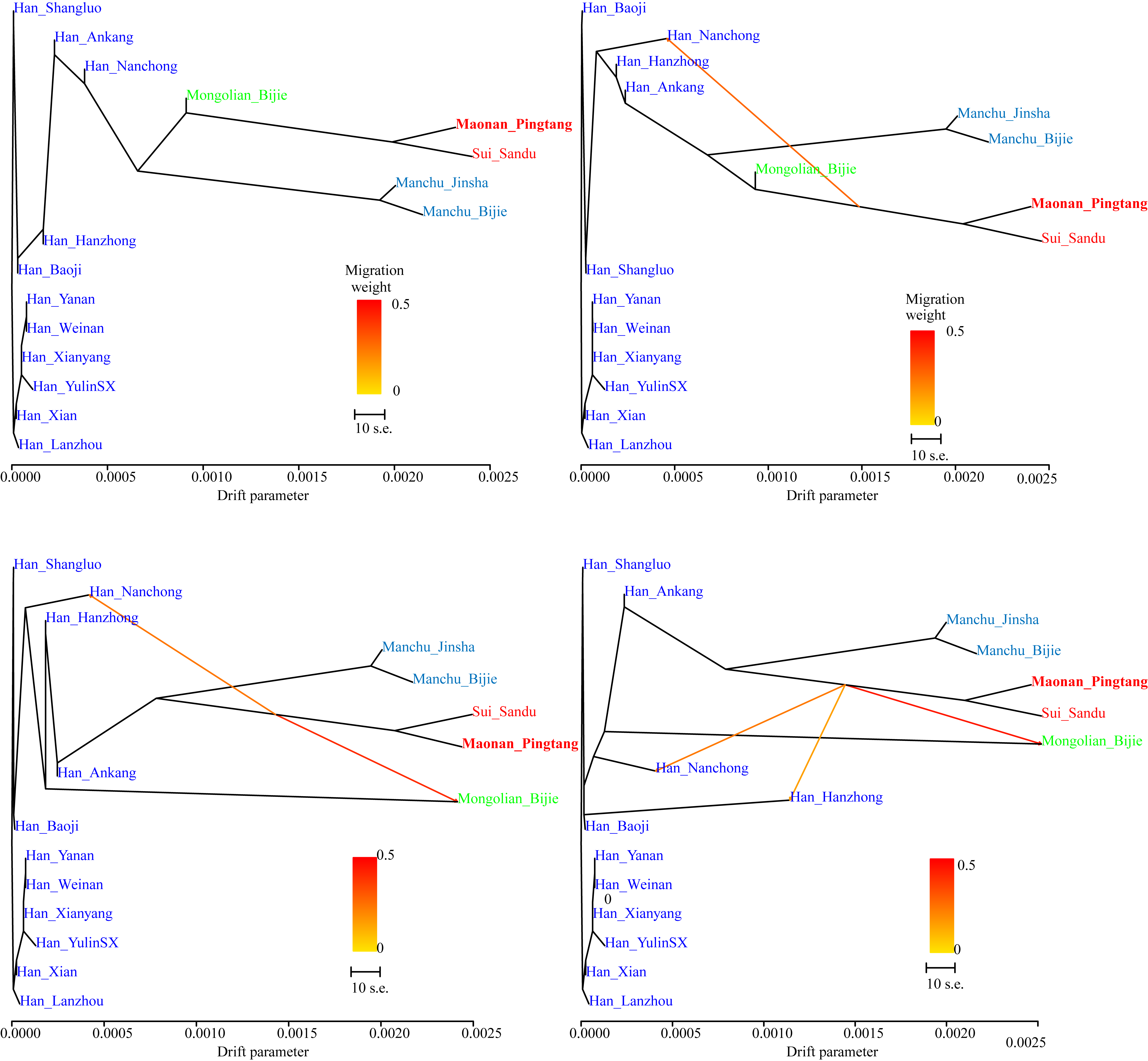

Supplement: Supplementary file 4 [file Image3.TIF]

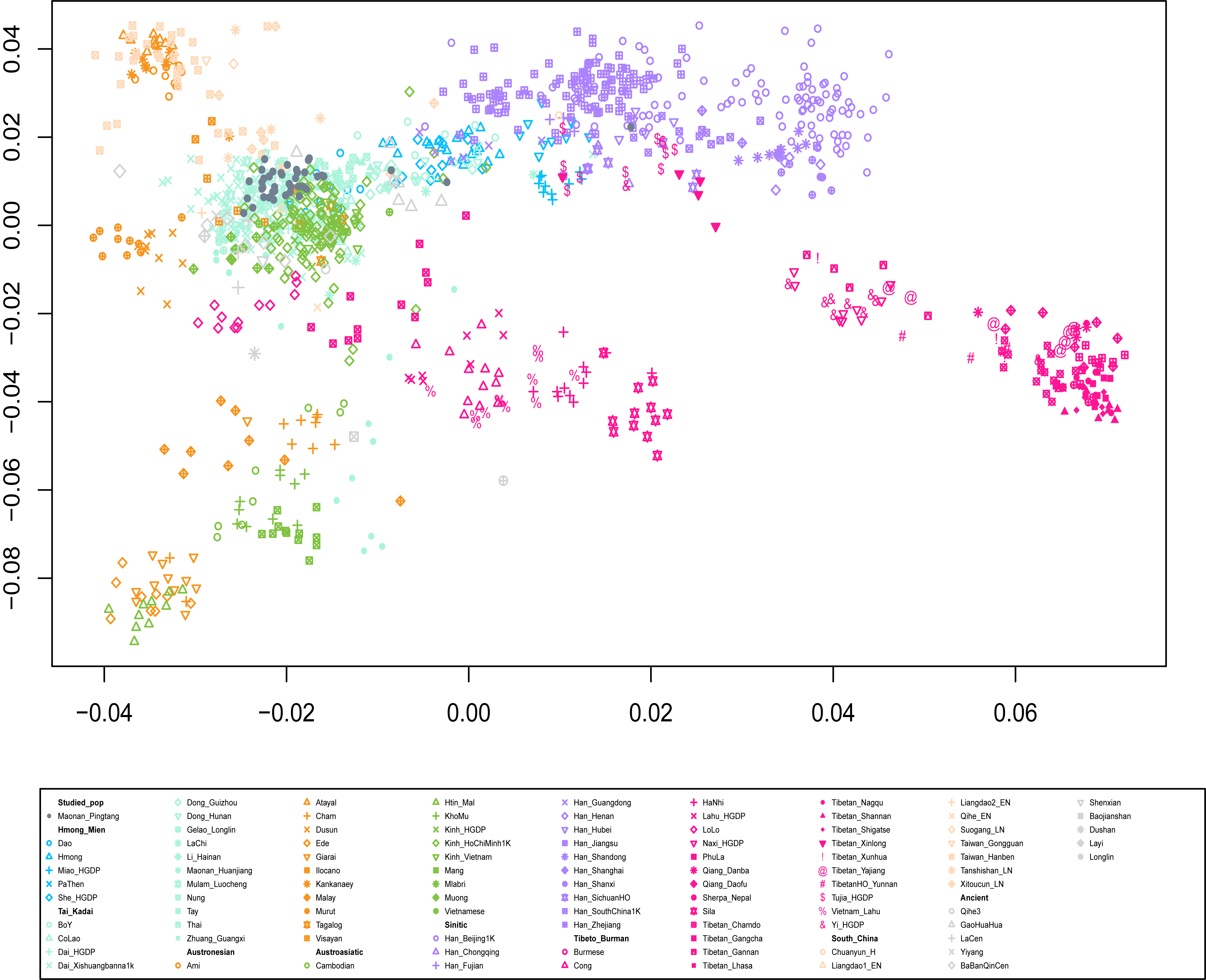

Supplement: Supplementary file 5 [file Image2.TIF]
